# Effect of Heat Shock Treatment on the Virulence of Grass Carp Reovirus in Rare Minnow *Gobiocypris rarus*

**DOI:** 10.3390/v16060921

**Published:** 2024-06-05

**Authors:** Qinwei Ni, Yanchang Fan, Simin Xiao, Liqun Lu

**Affiliations:** 1 National Pathogen Collection Center for Aquatic Animals, Shanghai Ocean University, Shanghai 201306, China; m210100310@st.shou.edu.cn (Q.N.); m220100204@st.shou.edu.cn (Y.F.); m210100361@st.shou.edu.cn (S.X.); 2 Key Laboratory of Agriculture Ministry for Freshwater Aquatic Genetic Resources, Shanghai Ocean University, Shanghai 201306, China; 3 Department of Aquatic Medicine, College of Fisheries and Life Science, Shanghai Ocean University, Shanghai 201306, China

**Keywords:** grass carp reovirus, heat shock response, HSP70, virulence, rare minnow *Gobiocypris rarus*

## Abstract

The mode and outcome of fish–virus interactions are influenced by many abiotic factors, among which water temperature is especially important in poikilothermic fish. Rare minnow *Gobiocypris rarus* is a eurythermal small cyprinid fish that is sensitive to infection with genotype II grass carp reovirus (GCRV). HSP70, a conservative and key player in heat shock response, is previously identified as an induced pro-viral factor during GCRV infection in vitro. Here, rare minnow was subjected to heat shock treatment (HST), 1 h treatment at 32 °C followed by reverting to a normal temperature of 24 °C, and subsequently challenged with GCRV-II at a dosage of 1 × LD_50_. The effect of HST on GCRV virulence in vivo was evaluated by calculating virus-associated mortality and viral load in both dead and survival fish. The results revealed that HST enhanced the mortality of rare minnow infected with GCRV; the fact that viral loads in the tissue samples of HST-treated fish were significantly higher than those in samples of the control group at 6, 8 d p.i. reflected a faster infection process due to HST. Quantitative gene expression analysis was further employed to show that the expression levels of *Hsp70* in intestine and liver tissues from the HST group declined faster than muscle tissue after HST. HST W/O GCRV challenge upregulated proinflammatory cytokines such as *MyD88* and *Nf-κB*, which was in consistence with the inflammation observed in histopathological analysis. This study shed light on the complexity of the interaction between fish abiotic and biotic stress response, which suggested that HST, an abiotic stress, could enhance the virulence of GCRV in *Gobiocypris rarus* that involved modulating the gene expression of host heat shock, as well as a pro-inflammatory response.

## 1. Introduction

Grass carp reovirus (GCRV) in the genus *Aquareovirus* has been known for causing viral hemorrhagic disease in cultured grass carp *Ctenopharyngodon idella*, which was first identified from grass carp in China [1]. The GCRV strains isolated and characterized in the last decades can be classified into three major genotypes, designated as groups GCRV I, II, and III, which are represented individually by the strain GCRV-873, GCRV-HZ08, and GCRV-104 [2]. GCRV-JX02, which belongs to GCRV-II, was isolated in the diseased grass carp sample, and GCRV-JX02 infection has already been quantitatively detected via in vivo and in vitro characterization in our previous report [3]. According to the VP6 protein sequence of GCRV, the sequence similarity between different genotypes was less than 20% [4]. Although GCRV-II is the predominant genotype causing epidemics and economic losses throughout China in recent years, the infection of other genotypes might occur in the field sporadically [5]. In the case of natural infection, the amount of virus is higher in the trunk kidney, gill and spleen, but the hepatopancreas, intestine and muscle may be the main target organs of GCRV invasion in artificial injection infection [6]. Rare minnow *Gobiocypris rarus* is an eurythermal small cyprinid fish distributed in the natural water of southwest of China, which recently emerged as a superior laboratory model fish for the virology of GCRV-II, largely due to its relatively small size (5–6 cm), short reproductive cycle (3–4 months) and sensitivity to GCRV-II challenge with the rapid onset of hemorrhagic symptoms [7].

The growth rate of aquatic animals is generally temperature-dependent. Interestingly, the occurrence of infectious diseases also displays seasonality and temperature dependency throughout the world [8]. Highly diverse and conserved heat shock proteins (HSPs) are initially identified as molecular chaperones displaying housekeeping functions such as protein folding, assembly, transport and degradation, and have been later shown to have multiple other functions involving cell signaling pathways, transcription regulation and immune response [9]. HSPs inside the cells not only help maintain general cellular homeostasis, but are also induced or modulated during stress conditions to function as immune effectors and acute-phase proteins [10]. HSPs are divided into different classes: small HSPs, HSP30, HSP60, HSP70, HSP90 and HSP100; among them, HSP70 is a key molecular chaperone and the most studied HSP that is overexpressed in the cell in response to the stress of various origin, including pathogen infection [11]. Heat shock response (HSR) has been implicated in viral replication cycles through various HSPs [12]. HSP70 has been especially shown to be involved in the formation of viral replication transcript complexes and regulating viral replication in different stages of each individual viral life cycle [13]. It is reasonable to speculate that HSR should play outstanding roles in aquatic ectotherms during temperature-fluctuation-induced viral epidemics. Additionally, the induction of HSR is not limited to temperature change, and the selective expression of HSPs in cells can be induced by many other stresses such as heavy metals, UV irradiation, osmotic stress and viral infection [14]. Thus, targeting HSR might provide a broad-spectrum strategy against various fish viral infections.

HSP70 and HSP90 have been shown to be induced by and actively promote GCRV infection in vitro [15,16]. Neither vaccine nor medicine is commercially available for farmers to control GCRV-II at present. In vitro studies have shown that heat shock treatment (HST) enhances the infection of GCRV, and the pro-viral effects of HSR could be counteracted in vitro by quercetin, a natural HSP70 inhibitor [17]. An in vivo study has validated that quercetin can effectively increase the survival rate of rare minnow *Gobiocypris rarus* from GCRV-II infection [18]. HSR is thus considered a potential target for developing novel anti-GCRV therapy. However, the effect of HST in rare minnow on GCRV-II virulence in vivo still needs to be clarified. Evidence on the correlation of the up-regulated expression profiles of HSP70 in fish tissues with enhanced viral replication levels during HST should help in the clarification of the role of HST in disease occurrence, which would pave the way for the application of quercetin as a potential drug against GCRV infection.

Here, a rare minnow/GCRV-II infection system was utilized to monitor the infection and pathogenesis of GCRV in vivo following HST. HST was demonstrated to enhance the virulence of GCRV, which involved regulating the fish gene expressions of both heat shock and pro-inflammatory responses.

## 2. Materials and Methods

### 2.1. Virus and Fish

The GCRV-JX02 virus used in this study was a laboratory stock [3]. The rare minnow *Gobiocypris rarus* (45 mm ± 5 mm, 0.8 ± 0.1 g) was provided by the Institute of Hydrobiology, Chinese Academy of Sciences, and bred in the fish house of the National Aquatic Animal Pathogen Collection Center of Shanghai Ocean University. The fish were cultured under controlled temperature conditions (24 ± 1 °C). All experiments were performed according to the guidance of the Care and Use of Laboratory Animals in China.

### 2.2. Heat Shock Treatment and Sample Collection

In the heat shock experiment, a group of 24 fish were transferred from the acclimation tank (24 °C) to a tank preheated and maintained at 32 °C. The livers, intestines and muscles of three individuals were collected randomly from non-shocked fish (control) and fish undergoing heat stress in various time periods ranging from 0.5 h to 12 h. All samples were immediately frozen at −80 °C until further analysis.

### 2.3. Semi-Lethal Dose (LD_50_)

Rare minnow was acclimated for two weeks in glass tanks and fed daily with artificial feed. The water temperature was maintained at approximately 24 °C. Healthy rare minnow was injected intraperitoneally with approximately 100 μL of the original virus stock. Rare minnows with obvious signs of infection were observed and collected daily and stored at −80 °C. Dead rare minnows were collected and the tissue was ground with homogenizer and filtered with a 0.22 μm sterile syringe filler by centrifugation with PBS. The virus solution was diluted in five gradients and injected intraperitoneally into healthy rare minnows with approximately 100 μL of virus at three parallel concentrations each, and the mortality of rare minnows at different virus concentrations was observed and recorded daily. The LD_50_ referred to the dosage that resulted in 50% death of the tested rare minnows. The LD_50_ for rare minnow mortality was calculated using probabilistic unit regression method with SPSS 26.0.

### 2.4. Virus Challenge and Survival Rate

To elucidate the effects of HST on GCRV-JX02 and proinflammatory gene expression in rare minnows, we randomly divided rare minnows into heat-shocked and non-shocked control groups, with 40 fish per group. Fish in the heat shocked group were placed in the acclimation tank (24 °C) and subjected to short-term heat exposure for 1 h at 32 ± 1 °C; then, they were transferred back to the tank (24 °C). Fish in the non-shocked group were kept at room temperature (24 °C). For infection, virus was diluted to 1 × LD_50_ in 100 µL of PBS and injected intraperitoneally into healthy rare minnows. The liver, intestine and muscle tissues of three individuals were collected from both groups. All samples were immediately frozen at −80 °C until further analysis.

The experiment of virulence of GCRV-JX02 in rare minnows was conducted as previously described with slight modifications. Twenty-eight fish were divided into either the heat-shocked or non-shocked control group, and the mortality of rare minnows was observed and recorded daily.

### 2.5. Histopathological Analysis

The liver, intestine and muscle tissues were harvested and fixed with 4% paraformaldehyde fixation solution at room temperature for 24 h. The serial tissue sections were cut to 3 µm thickness after embedding in paraffin. The slides were stained with hematoxylin and eosin (H&E) and examined by light microscopy.

### 2.6. RNA Isolation and RT-qPCR

Total RNA was extracted from the liver, intestine and muscle tissues homogenized with TRIzol (Invitrogen, Shanghai, China), according to the manufacturer’s instructions. RNA was reversely transcribed into cDNA by PrimeScriptTM II 1st Strand cDNA Synthesis Kit (TaKaRa, Dalian, China). M6 genomic fragment of GCRV-II was targeted to monitor the genome replication level using qRT-PCR with TB Green^®^ Premix Ex Taq™ II (TaKaRa). The following primers were used for the qPCR of GCRV-JX02: forward primer, 5′-GCGTCACACCTTCGACCATA-3′; and reverse primer, 5′-TTACGGGCCGGAAATAGTGG-3′. The standard curve was constructed to calculate the copy number of the viral genome copies. Standard RNA was transcribed in vitro using the M6 gene of GCRV-II as the template, and standard RNA was serially diluted 10-fold to construct a standard curve.

The expression levels of *Hsp70* and selected immune genes were quantified by qPCR with *β-actin* serving as an internal control gene for cDNA normalization. The expression levels were calculated by 2^−ΔΔCT^, and the value stood for an n-fold difference relative to the calibrator. All data were given in terms of relative mRNA expressed as the means ± S.E. The primers used are listed in Table 1.

### 2.7. Western Blot

The liver, intestine and muscle tissues were lysed in a RIPA buffer on ice, and protein extracts were separated by 10% SDS-PAGE followed by transferring onto PVDF membranes. Then, the membranes were blocked with 5% skim milk powder at room temperature for 2 h. The concentrations of primary rabbit antibody against HSP70 (Cys-RGGSGAASQGPTIEEVD) were 0.96 mg/mL and 1:3000 diluted with 2.5% skim milk. The PVDF membranes subsequently incubated with primary antibody HSP70 at 4 °C for 12 h. After stringent washing, the PVDF membranes were incubated with commercial HRP-conjugated goat anti-rabbit IgG goat polyclonal antibody (HUABIO, Hangzhou, China) at room temperature for 2 h. For detection, BeyoECL Plus (Beyotime, Shanghai, China) and ChemiDocTM Imaging System were used to detect the blot according to the manufacturer’s instructions.

### 2.8. Statistical Analysis

The differences between the data of different groups was compared by a one-way analysis of variance and Student’s t-test using GraphPad Prism 8.0.2 software. A *p*-value lower or equal to 0.05 was considered statistically significant, and the asterisks indicate significant differences compared to the control group (* *p* < 0.05; ** *p* < 0.01; *** *p* < 0.001; **** *p* < 0.0001). Results are the mean ± standard deviation (SD) (n = 3).

## 3. Results

### 3.1. Optimization of Heat Shock Treatment (HST) Process Condition for Rare Minnows with Hsp70 as an Indicator Gene

**Figure 1:**
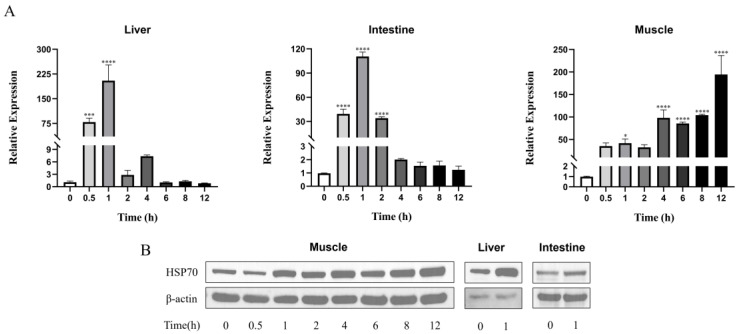
HST increased the expression of HSP70 in rare minnows. Rare minnows were exposed to 32 °C for 12 h and sacrificed at 0, 0.5, 1, 2, 4, 6, 8 and 12 h after HST for tissues collection. (**A**) *Hsp70* expression level in the liver, intestine and muscle tissues measured by qPCR. (**B**) HSP70 expression in the liver, intestine and muscle tissues by a Western blot assay. The muscle tissues were extracted at various time points as indicated in the figure, while the liver and intestine tissues were extracted at 1 h post HST. Gene expression was normalized with *β-actin* as an internal standard and then compared with the 0 h group (* *p* < 0.05; *** *p* < 0.001; **** *p* < 0.0001).

The conservative HSP70 chaperone machinery is a key component of the HSR, which is characterized by its immediate induction following a sudden increase in temperature [19]. The rare minnows cultured at 24 °C were transferred to a fish tank with a water temperature of 32 °C. To optimize the longevity of the HST process, the *Hsp70* expression level was monitored at 0, 0.5, 1, 2, 4, 6, 8 and 12 h following the transfer by randomly sacrificing three fish for tissue extraction (Figure 1). The level of *Hsp70* gene expression rose steadily over time in muscle tissue, while the expression of *Hsp70* reached the highest level at 1 h following HST in both fish liver and intestine tissues (Figure 1A). Translational expression analysis by a Western blot assay confirmed the up-regulated protein level of HSP70 in these tissues following HST (Figure 1B). Thus, 1 h treatment at 32 °C is enough to stimulate the rare minnow HSR in our system.

**Figure 2:**
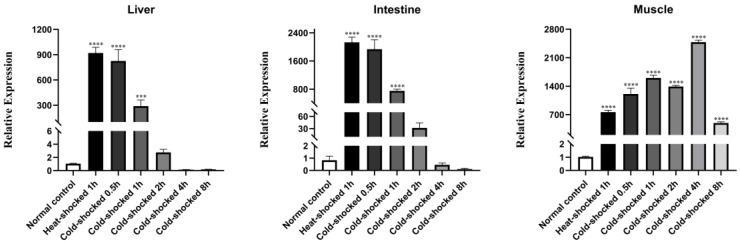
Effect of HST of 1 h followed by reverting to normal temperature on *Hsp70* expression. After heat shock for 1 h, rare minnows were transferred to a tank at 24 °C, and sacrificed at 0.5, 1, 2, 4 and 8 h after cold shock for tissue collection. *Hsp70* expressions in the liver, intestine and muscle were measured by qPCR. Gene expression was normalized with *β-actin* as an internal standard and then compared with the normal control group (*** *p* < 0.001; **** *p* < 0.0001).

**Figure 3:**
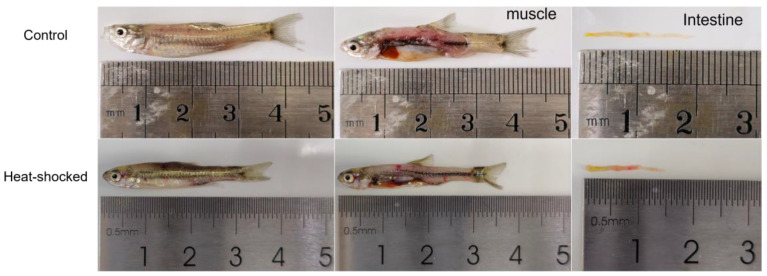
Infection of rare minnows W/O HST resulted in unaided bleeding symptoms in the muscle and intestine tissues.

The fact that rare minnows were transferred back to a tank with a normal water temperature of 24 °C following HST also suggested that the fish were undergoing a cold shock treatment (CST). It remained to clarify the effect of CST on *Hsp70* expression, which was known to decrease *Hsp70* in human cells [20,21]. For this purpose, at different time points following the CST, rare minnows were sampled for tissue extraction and gene expression analysis. The expression level of *Hsp70* in the liver and intestine tissues immediately decreased, and could reach a significantly lower level than that of normal fish at 4 h post CST; on the contrary, the expression of *Hsp70* continued to rise until 8 h post CST in muscle tissues (Figure 2). Thus, CST seemed to negatively regulate the expression of *Hsp70* in the intestine and liver, while positively regulating *Hsp70* expression in the muscle. Figure 3 indicates that the infection of rare minnows with a lethal dosage of GCRV resulted in extensive bleeding symptoms in the muscle and intestine, independent of HST. Considering that muscle is the unaided target tissue of GCRV in rare minnows displaying bleeding symptoms [18], 1 h treatment at 32 °C followed by recovery at 24 °C was selected as the optimal HST procedure for rare minnows in this study.

### 3.2. Heat Shock Treatment Enhances the Virulence of GCRV in Rare Minnows

To quantify the effect of HST on the virulence of GCRV, LD_50_ of GCRV was determined by injecting serious dilutions of virus stock solution into healthy rare minnows. Table 2 shows the mortality of rare minnows in response to different concentration of injected virus copy numbers. The SPSS 26.0 software was employed to calculate a LD_50_ value of 206,910.682 copies/100 μL with a 95% confidence interval of 48,313.052~439,915.36.

**Figure 4:**
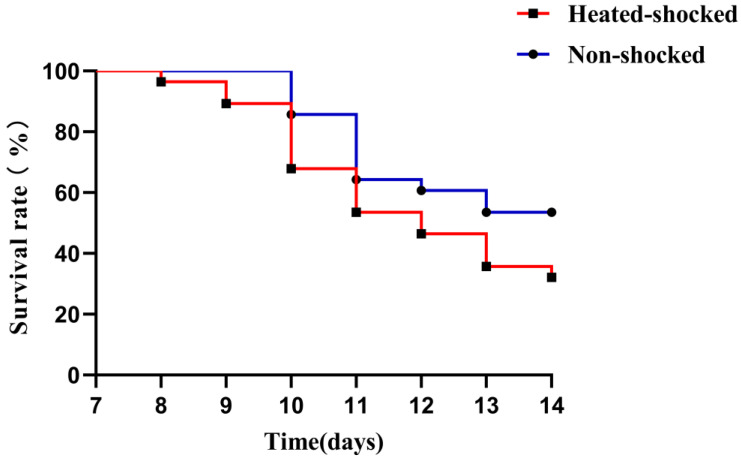
Short-term heat shock reduced the survival rate of rare minnow infected with GCRV-JX02. Rare minnows were randomly divided into heat-shocked and non-shocked groups. Rare minnows in the heat shock group were exposed to 32 °C for 1 h before being infected with 1 × LD50 GCRV-JX02 intraperitoneally. The survival rate of each group was monitored for 14 days post infection.

In the following animal challenge experiments, fish were exposed to 32 °C for 1 h for HST before being challenged with 1 × LD_50_ GCRV through intraperitoneal injection. The survival curve (Figure 4) indicated that the fish from the HST group began to die at Day 8, which was 2 days earlier than the control group. The overall mortality of the HST group was significantly higher than that of the control fish, resulting a lower survival rate of 32.14% in contrast to the control of 53.57%. This result suggested that HST enhanced the lethality of GCRV-JX02 in rare minnow.

**Figure 5:**
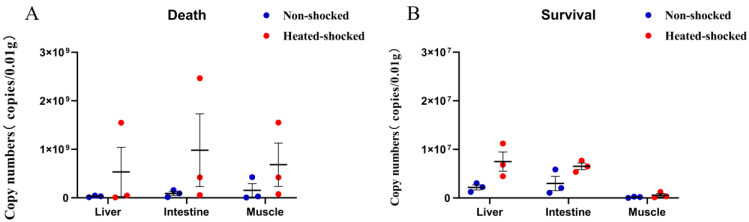
HST enhanced viral replication in dead rare minnows. Rare minnows in the heat shock group were exposed to 32 °C for 1 h before being infected with LD50 GCRV-JX02 intraperitoneally. (**A**) Viral replication in dead samples quantitated by real time RT-PCR. Rare minnows were sacrificed immediately after death. (**B**) Viral replication in surviving rare minnows quantitated by real-time PCR. Fish of the survival group were sacrificed and sampled at Day 14 post infection for the harvesting of the liver, intestine and muscle tissues.

To verify the successful infection of GCRV in rare minnows in the above animal challenging experiments, three dead fish together with three surviving fish were randomly picked from either the HST or control group, and the viral genome copy numbers in the muscle, intestine and liver tissues was quantitatively calculated. Viral replication was confirmed in all tested samples, and the viral titer in the dead fish was 50–100 times higher than that in the surviving fish (Figure 5). In the dead samples, the viral replication level of the HST group was found to be significantly higher than that of the control group (Figure 5A). In the survival samples, no significant difference of viral replication was detected between the HST and the control group (Figure 5B).

**Figure 6:**
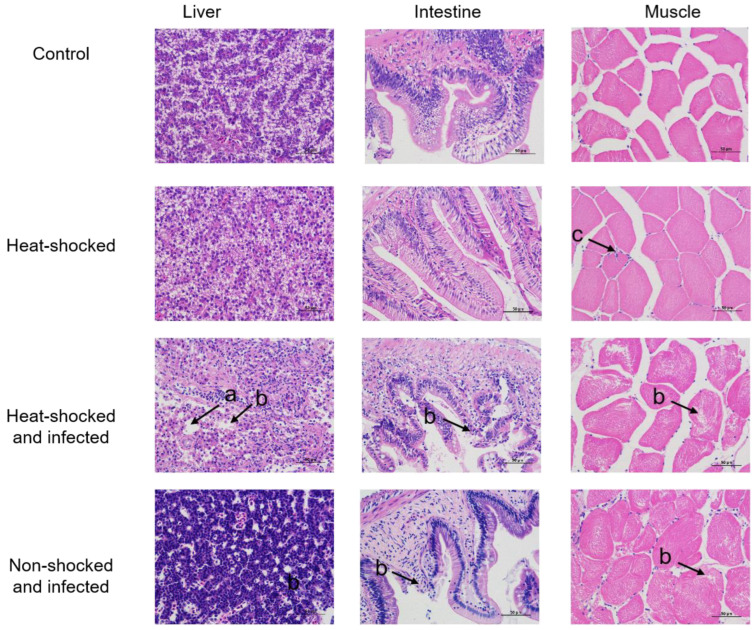
Histological differences in the liver, intestine and muscle of the infected rare minnows W/O HST and control fish. The samples of liver, intestine and muscle from the infected fish and the control fish (healthy fish) were fixed, embedded, sectioned and stained with hematoxylin and eosin (HE). The slides were examined via light microscopy. (a) Vacuolation marked by an arrow; (b) the arrow shows extensive focal necrotic lesions; (c) the arrow shows the inflammatory edema of muscle cells.

Furthermore, the dead fish collected at 14 d p.i. were randomly picked for pathological examination of the liver, intestine and muscle tissues by a HE staining assay. As shown in Figure 6, extensive tissue hydrolysis and tissue vacuole were detected in GCRV-infected rare minnows in either the liver or intestine; on the other hand, inflammation and edema dominated in the muscle tissue. Overall, the tissue injury of the HST group was heavier than that of the control group. Tissue section analysis did reveal the inflammatory-enhancement effect of HST.

### 3.3. Pro-Viral Effect of HST in Rare Minnows Correlated with Enhanced Proinflammatory Gene Expression

**Figure 7:**
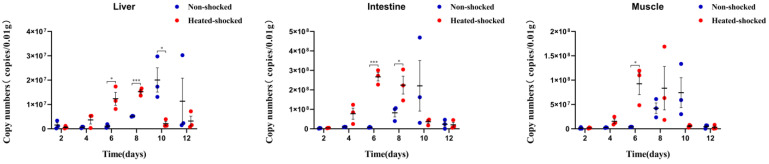
HST accelerated the infection of GCRV in rare minnows. Rare minnows in the heat shock group were exposed to 32 °C for 1 h before being infected with 1 LD50 GCRV-JX02 intraperitoneally. Fish were sacrificed at Days 2, 4, 6, 8, 10 and 12 post infection for the harvesting of the liver, intestine and muscle tissues. The genome copy numbers of GCRV-JX02 were determined by qPCR. The copy numbers were normalized with weight as the standard (* *p* < 0.05; *** *p* < 0.001).

In the surviving fish collected at 14 d p.i., HST did not promote the replication of GCRV, which was in contrast to that in the dead fish (Figure 5). It is speculated that the anti-viral response of surviving fish might contribute to their survival. To clarify this, an in vivo replication efficiency of GCRV in alive rare minnows was systematically compared between the HST and control groups from day 1 p.i. Time course study (Figure 7) indicated that the viral titers in the tested HST samples were higher than the control fish samples in every tested time points until the eighth day. Particularly, the differences of viral titers was significant at 6, 8 d p.i., while the viral load of the HST group could be lower than the control group in the tested tissues from 10 d p.i. Figure 7 suggests that the pro-viral effect of HST in rare minnow could be compromised by host response at late stages of GCRV infection, which might represent a recovery stage from viral invasion. The results correlated with the finding that mortality of the HST group only appeared before 10 d p.i. in Figure 4. These results implied that HST accelerated the virus infection and increased mortality in rare minnows.

**Figure 8:**
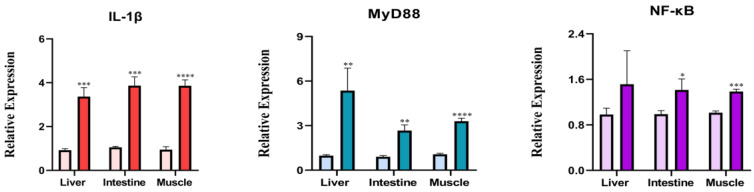
One h HST-modulated proinflammation gene expression. Rare minnows in the heat shock group were exposed to 32 °C for 1 h. *IL-1β*, *MyD88* and *NK-κB* expressions were determined by qPCR. Gene expression was normalized with *β-actin* as an internal standard and then compared with the normal control group (* *p* < 0.05; ** *p* < 0.01; *** *p* < 0.001; **** *p* < 0.0001).

**Figure 9:**
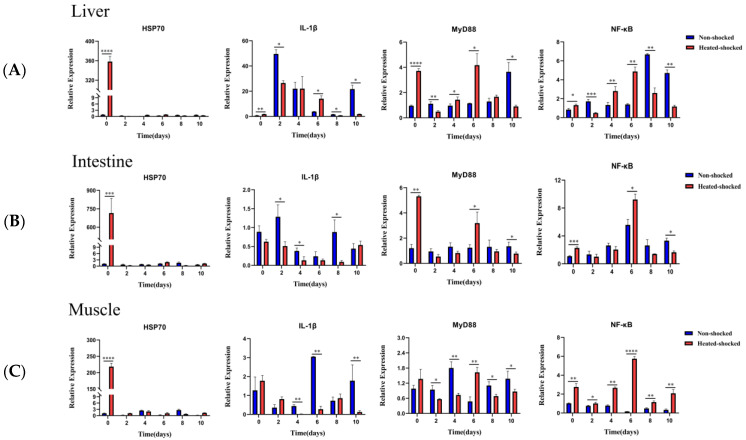
HST coupled with GCRV challenge modulated proinflammation gene expression. Rare minnows in the heat shock group were exposed to 32 °C for 1 h. The liver (**A**), intestine (**B**) and muscle (**C**) tissues were collected at 0, 2, 4, 6, 8 and 10 days after the virus challenge. *HSP70*, *IL-1β*, *MyD88* and *NK-κB* expressions were determined by qPCR. Gene expression was normalized with *β-actin* as an internal standard and then compared with the normal control group (* *p* < 0.05; ** *p* < 0.01; *** *p* < 0.001; **** *p* < 0.0001).

Fish immune response also played a key role for fish survival. To probe the effect of HST alone on host immune response, the levels of selected inflammatory genes were monitored by quantitative real-time PCR analysis in rare minnows without viral challenge. Compared with the control rare minnows, 1h HST increased the mRNA levels of *IL-1β*, *MyD88* and *NF-κB* (Figure 8). In order to further elucidate the effect of HST on the viral infection outcome in rare minnows, the mRNA levels of *Hsp70*, *IL-1β*, *MyD88* and *NF-κB* in rare minnows were systematically monitored following GCRV challenge at Day 0, 2, 4, 6, 8 and 10 p.i. (Figure 9). In liver (Figure 9A), intestinal (Figure 9B) and muscle (Figure 9C) tissues, 1 h HST process resulted in dramatically up-regulated *Hsp70* at the starting time point (day 0), but the expression of *Hsp70* in the HST group reduced to a normal and low level since day 2. HST coupled with GCRV challenge did up-regulate the expression of *MyD88* and *NF-κB* in all tested tissues at day 0 and 6 p.i.; *IL-1β* was not up-regulated significantly at day 0 and expressed at a comparably lower level than that of the control fish in the tested tissues (except for Day 6 p.i. in liver). In comparison with the result in Figure 8, HST coupled with GCRV challenge appeared to have a similar impact on rare minnow gene expression of *MyD88* and *NF-κB* as HST treatment alone.

The above primary results implied that short-term HST induced the expressions of *HSP70* and pro-inflammatory cytokines *MyD88* and *NF-κB* W/O GCRV challenge. Thus, modulating the immune gene expression profile might also be involved in determining the infection outcome of individual rare minnows due to HST.

## 4. Discussion

GCRV has served as a prototype virus of Aquareoviruses in recent years due to its pathogenic nature and causing heavy loss in cultured grass carp [22]. Although there is no experimental SPF grass carp with a clear genetic background for characterizing GCRV, rare minnow *Gobiocypris rarus*, a model fish in the field of toxicology and genetics with a clear genetic background, is known to be the natural host of GCRV [18]. Previously, all HSP70 inhibitors have been shown to be effective in suppressing GCRV replication in vitro [23]. Similarly, we reported that the pro-viral effect of HSR could be counteracted in vitro by HSP70 inhibitor quercetin [17]. The pro-viral effect of HST in vivo is in consistence with previous in vitro analysis. The HST of rare minnows induced HSP70 expression in all tested tissues (Figure 1 and Figure 2). In dead fish, the viral load was significantly higher than that of the surviving fish, which was in consistence with previous results that suggested that dosage–dependent mortality could be achieved during GCRV challenge in rare minnows [18]. Most viruses need cellular chaperones during their life cycle; therefore, HSP70 chaperones, as central components of the cellular chaperone network, are frequently recruited by viruses [13]. HSP70 was shown to co-localize with GCRV dsRNA in the viral synthesis factory of infected grass carp cells to facilitate viral replication [17]. Induced HSP70 or HSP90 also locates onto the cellular membrane to facilitate GCRV attachment and infection [24,25]. However, we noticed that the HSP70 level could reverse back to a normal level after the HST process (Figure 2 and Figure 9), which suggested that HST only induced transit *Hsp70* up-regulation. This might explain why there was not much difference of viral titers during the late infection of rare minnows between the HST group and control fish. Maintaining high-level HSR for a long period did not seem to be a reasonable choice for aquatic animals. In a study to evaluate the impacts of heat wave on the survival of frogs, compromising the protection of HSP in frogs was indeed considered a strategy for the frogs to maintain a low-energy state to survival [26].

An inflammation response is critical for fish defense against viral infection. We observed that the HST group demonstrated stronger inflammation symptoms following GCRV challenge (Figure 3 and Figure 6). Pathological examination further indicated that HST alone caused significant inflammation in muscle tissue (Figure 6). The interactions of HSPs with signaling pathway regulators in immune cells suggest their crucial roles in inflammation responses. In zebrafish, *IL-1β* and *IL-6* transcripts were generally up-regulated with increased temperature of the secondary thermal challenge, whereas IFN-1 transcripts were up-regulated in the spleen, but down-regulated in the gills, along with MH class I [27]. HSP90 inhibition robustly suppressed TPA-induced inflammation by targeting key proinflammatory cytokines and signaling pathways [28]. Heat stress caused extreme small intestine damage enhanced oxidative stress and activated MAPK signaling pathways [29]. Nf-κB is a major transcriptional activator of inflammatory mediators such as cytokines, and TLR-MyD88-NFkB serves as one of the major intracellular mechanisms to defend against viral infections of mammalian cells [30]. A study on the transcriptional levels of *MyD88* and *Nf-κB* in the HST group W/O GCRV challenge indicated that HST resulted in a proinflammatory effect in both GCRV-infected and mock-infected rare minnows (Figure 8 and Figure 9). An inflammatory reaction might explain the increased mortality and is also responsible for an efficient anti-viral response in surviving fish.

Muscle tissue was the only tissue that demonstrated obvious inflammation following HST treatment without GCRV challenge upon pathological examination (Figure 6). One interesting discovery in this study is the fact that HST demonstrated similar impacts on *Hsp70* expression patterns in the liver and intestine, while a lagged rising and falling *Hsp70* was recorded for muscle tissue (Figure 1 and Figure 2), which suggests that muscle tissue could undergo stronger heat shock stress than other tissues. Correlated with this, the proinflammation gene expression patterns in muscle following HST demonstrated certain differences from the liver and intestine tissues (Figure 8 and Figure 9). It was also noted that the surviving fish that were GCRV-challenged demonstrated no observable symptom and grew normally in this study. Recently, GCRV was reported to remain latent in grass carp and can reactivate in response to environment stimulation [25]. Thus, as GCRV carriers, the surviving fish might serve as a virus source for the next epidemic. This study did not exclude the possibility that an adaptive immune response, together with the innate immune response, might eliminate GCRV from the surviving fish. However, primary evidence indicated that the surviving rare minnows could pass the virus via vertical transmission [31], which supported the idea that GCRV could remain latent in host fish.

## 5. Conclusions

We employed a rare minnow/genotype II GCRV infection system to show that HST increased the virulence of GCRV by increasing the mortality and accelerating pathogenesis in vivo. Our study suggested that HST could confer the sensitivity of rare minnows to GCRV-II infection and promote viral pathogenesis. The findings should contribute to the understanding of the temperature dependency of GCRV-caused hemorrhagic disease in grass carp cultivation, as well as pave the way for developing novel and broad-spectrum anti-viral strategies by targeting the HSR. The study highlighted the roles of temperature-fluctuation-induced inflammation and pro-viral effect of HSP during viral pathogenesis in poikilothermic fish.

## Figures and Tables

**Table 1:** Primers used in this study.

Primer Name	Sequence (5′–3′)
GCRV-JX02	F:5′-GCGTCACACCTTCGACCATA-3′ R: 5′-TTACGGGCCGGAAATAGTGG-3′
β-actin	F:5′-CTATGTTGGTGACGAGGCTCA-3′ R: 5′-CCCAGTTGGTGACAATACCG-3′
HSP70	F: 5′-GTGTCCATCCTGACCATTGAAG-3′ R: 5′-GTGCCCTCTTGTTCTGACTGAT-3′
IL-1β	F: 5′-TGATGAGATGGACTGCCCTG-3′ R: 5′-TGTCCGTCTCTCAGCGTCAC-3′
MyD88	F: 5′-GGTGGTAATTTCCGATGA-3′ R: 5′-GTAGACAACAGGGATAAGG-3′
NF-kB	F: 5′-AACTCAGTCAGGCTCCATTGC-3′ R: 5′-GACAGTGCTCTCCGTCTTTCC-3′

**Table 2:** Mortality of rare minnows challenged with different titers of GCRV-JX02.

Group	Copies of Virus (100 μL)	lg Copies	Total Number of Fish	Cumulative Deaths	Mortality
1	1,869,085	6.272	10	9	90%
2	422,259	5.626	10	6	60%
3	345,391	5.538	10	6	60%
4	152,582	5.184	10	5	50%
5	88,753	4.948	10	3	30%

## Data Availability

Data are contained within the article.
